# DIFFERENCES IN PATIENTS WHO FAIL A COGNITIVE SCREEN IN PRIMARY CARE VS. THOSE WHO “ALMOST FAIL”

**DOI:** 10.1093/geroni/igae098.4160

**Published:** 2024-12-31

**Authors:** Anna Higbie, Christina Baucco, Anthony Perkins, Nicole Fowler

**Affiliations:** Regenstrief Institute, Indianapolis, Indiana, United States; Indiana University, Indianapolis, Indiana, United States; Indiana University, Indianapolis, Indiana, United States; Indiana University, Indianapolis, Indiana, United States

## Abstract

In primary care, short cognitive screening tools are used to measure potential cognitive impairment. These tools have been validated in patients aged 65+ and have published cut points to determine if further assessment is needed. We conducted a secondary analysis of data from the Caregivers Outcomes in Alzheimer’s Disease (COADS) trial to compare characteristics of patients who screened positive using established cut points versus those who scored one point away. Screening tests included the Mini-Cog, MIS-T, and MIS-T & Virtual Clock Draw. We also compared mild cognitive impairment (MCI) and Alzheimer’s Disease and Related Dementias (ADRD) diagnoses between the two groups at 24 months. Among the sample, n=44 failed based on standard cut points, and n=94 scored one point from fail. There were no significant differences in the age, gender, or Charlson Comorbidity Index (CCI) between the groups. Patients one point from failing were significantly more likely to have more education and report daily in-person interactions (p=0.013 and p=0.020). At 24 months, no significant differences in the rate of MCI or ADRD diagnoses were found in electronic medical records. Although the group who had a positive screen at baseline had statistically significant differences in cognitive impairment at 24 months, as measured by the IQCODE (4.4 times more likely to score above 3.6, p=0.009, with higher cognitive decline indicated by their IQCODE scores, p=0.014). These findings suggest better performance on cognitive screeners is associated with reduced cognitive decline over time, underscoring the importance of early detection for at-risk populations.

